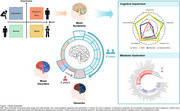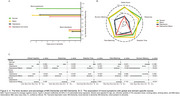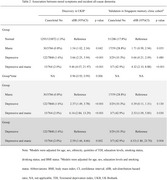# Mood symptoms, mood disorders, and dementia incidence in multi‐regional multi‐ethnic adults

**DOI:** 10.1002/alz70857_097364

**Published:** 2025-12-24

**Authors:** Haoran Zhang, Yingqi Liao, Haoxuan Wen, Ting Pang, Xuhao Zhao, Wanheng Zhang, Xiaowen Lou, Christopher Chen, Zuyun Liu, Shaohua Hu, Xin Xu

**Affiliations:** ^1^ School of Public Health, the Second Affiliated Hospital of School of Medicine, Zhejiang University, Hangzhou, Zhejiang, China; ^2^ Nanhu Brain‐computer Interface institute, Hangzhou, Zhejiang, China; ^3^ Memory, Ageing, and Cognition Centre (MACC), Department of Pharmacology, Yong Loo Lin School of Medicine, National University of Singapore, Singapore, Singapore; ^4^ School of Public Health and the Second Affiliated Hospital of School of Medicine, Zhejiang University, Hangzhou, Zhejiang, China; ^5^ The Zhejiang Key Laboratory of Precision Psychiatry, Hangzhou, Zhejiang, China; ^6^ Department of Psychiatry, the First Affiliated Hospital, Zhejiang University School of Medicine, Hangzhou, Zhejiang, China; ^7^ School of Brain Science and Brian Medicine, and MOE Frontier Science Center for Brain Science and Brain‐Machine Integration, Zhejiang University School of Medicine, Hangzhou, Zhejiang, China; ^8^ Key Laboratory of Intelligent Preventive Medicine of Zhejiang Province, Hangzhou, Zhejiang, China

## Abstract

**Background:**

Mood disorders including depression and bipolar disorders have been linked to dementia. However, early manifestation of bipolar disorder, especially manic symptom, were easily overlooked. The present study aimed to investigate the association of midlife and late‐life mood symptoms, especially their comorbidity, with long‐term dementia incidence among multi‐regional and ethnic adults.

**Method:**

The study used UK Biobank as a discovery dataset and three Asian studies as validation datasets. Participants aged > 35 were included in the analysis. Individuals with diagnosed mood disorders and dementia were excluded at baseline. Baseline mood symptoms were classified as: normal, manic symptoms, depressive symptoms, and comorbidity of depressive and manic symptoms. Long‐term (12 years) incident mood disorders (depression, mania and bipolar) and dementia were diagnosed and recorded. Primary outcome was dementia incidence. Secondary outcomes were domain‐specific cognitive function and metabolomics. Fine‐Gray sub‐distribution hazard models and linear regression were used to estimate the associations of mood symptoms with dementia risk, cognitive function and selected metabolites.

**Result:**

The study included 142,670 UK and 1,610 Asian participants (mean [SD] age, 57.2 [8.2] and 70.5 [7.3] years, respectively). Mood symptoms were prevalent (11.4% and 31.2%) among 1462 (1.0%) and 74 (19.4%) who developed dementia during a mean follow‐up of 11.0 and 4.4 years in community and clinical settings, respectively. The average durations from mood symptoms and disorders to dementia onset were 7.5 and 1.7 years, respectively. Comorbidity of depressive and manic symptoms was associated with an earlier onset and a higher risk of developing dementia (sub‐distribution hazard ratios [sHR]=9.46, 95% confidence interval [CI]=4.07–21.97; and sHR=4.32, 95%CI=2.10–8.88; respectively), as compared to single symptom or none (on average 0.9 and 1.6 year earlier). Comorbidity of symptoms were associated with worse cognition (B=‐0.32; 95% CI=‐0.38–‐0.25), especially in reasoning and numeric memory, and an exacerbation of metabolic dysfunction, especially in fatty acids, lipoproteins and triglycerides.

**Conclusion:**

Mood symptoms were prevalent among incident dementia patients. Comorbidity of mood symptoms in midlife and late‐life could lead to a higher cumulative risk of dementia. Future studies warrant in‐depth investigation of distinct pathophysiological mechanisms.